# Army Veterinary Service

**Published:** 1890-01

**Authors:** R. S. Huidekoper

**Affiliations:** Vet.


### ARMY VETERINARY SERVICE.

By R. S. Huidekoper, M.D., Vet.

*An address before the Veterinary Medical Society of the Veterinary Department, University of Pennsylvania.*

Gentlemen :—After giving your President my assent to his request, that I should address you on "Army Veterinary Service," I turned to think of the form in which I should place it before you, and found myself in very much of a dilemma. To tell you of this service in other countries would require entering into many details of customs and habits not in conformity with our own mode of life ; to tell you of the want of a service in this country would scarcely be what you desire, and to tell you of all that a service should be, in the United States, would run me into a realm of imagination too Arcadian, to be politic, in the face of a public, of whom we desire much and of whom we dare to ask but little. There are members of your society before me, whom I hope to see in the United States army uniform, as Veterinarians, and as commissioned officers, before you have forgotten the hard benches and the habits of drudgery which accompany the efforts to improve your minds, which inconveniences in some analagous form or other, you will find necessary adjuncts to all your endeavours to secure reputation and success. I will, therefore, try to give you, in as brief a form as possible, an outline of what has been, is, and may be, concerning this subject, that you may think it over, enlarge it for yourselves in your own minds, and determine if you are willing to do the work and make the sacrifices that will be required to attain the end. Demand and supply are sooner or later correlative in all of the details of life, so, when we read, that Alexander the Great was obliged to halt his large army, because the horses feet were sore, we can assume that this great General at once called to his aid the most expert healer of horses to take charge of the maimed beasts, and from necessity formed the first Army Veterinary Service, but of the details of this we know nothing. We do know, however, that the disability of 10,000 horses interfered with the movements of a brave army, and inconvenienced the plans of one of the world's conquerors. Xénophon

(445 years, B. C.), by the same instinct which made him a celebrated warrior, learned that his horses must be cared for, and in his treatise on cavalry, adds a chapter on some of the diseases of the horse, so that we can look upon him, as the first recognized army veterinarian—with the rank of general. Five hundred years later, however, in the bosom of a more enlightened people, and by the authority of a more civilized and better educated ruler, the Emperor Augustus, the official care of injured military horses was ordered, and veterinary hospitals, called veterinarium, was established. Under the Emperor Diocletian (300 A. D.), veterinarians were found attached to the Roman army, and infirmaries and hospitals, for the treatment of sick horses are mentioned. It is at about this period that we find the first traces of shoeing in the Roman army, though it was long before known to the cavalrymen of Germany. Apsyrtus, Veterinarian to the Army of Constantine the Great (325,–400, A. D.) was a distinguished authority. He recognized the difference of glanders from distemper, (strangles), described periodic ophthalmia, tetanus, anthrax and other diseases. He recognized the contagious character of glanders and anthrax, and counseled the isolation of animals affected with these diseases. A hundred years later Theomnestus was the army veterinarian of King Theodoric.

In the middle ages we find frequent mention of veterinarians attached to the bodies of troops, who bristled at the heels of all the Kings and great nobles of Europe. First in Italy and in Spain, and later in France and Germany, the necessity of expert care of war horses was recognized, and we find mention of military veterinarians who were held in much esteem. Under the first Republic in France, Lafosse, who had formerly been Veterinarian at the Court of the King, was appointed Inspector General of the horses in the cavalry. In the first year after the foundation of the Alfort Veterinary School, officers from the armies of Austria, Prussia, and Denmark was enrolled amongst its students. In 1769, A. D., each cavalry regiment of the French army furnished one student to the school, and in 1774, the French Government established twenty free scholarships, for students who would agree to enter the army, after finishing their studies. Later the number was raised to forty. While the veterinary schools in France are in charge of the Department of Agriculture, the Department of War takes an active interest in them, and the army secures about one-half of the graduates each year. The end of the last century and the beginning of the present one, saw veterinary schools established in all of the countries of Europe, either directly by the government, or with government aid, except in England, where the schools have always been private enterprises. In the more peaceful countries, like Switzerland and Denmark, the schools are attached to the Departments of Agriculture, but receive support from the Departments of War, and furnish in return veterinarians to the army. In France and Prussia the schools have been in turn under the direction of the Minister of War and of Agriculture. In Prussia the army controls over nine-tenths of all of the students. Many of these enlist, and after carrying the musket for one year, are sent to the Berlin school in uniform, and under pay, where they receive their education at the expense of the government. For each six months at the school, the soldier has to serve one year as veterinarian in the army, so with the three-and-a-half years required at this school, the service is seven years. If a student fails in one-half-year examinations, and has to take an extra six months instruction, he is called on to serve his eighth year in the army before he can retire to the more profitable civil offices and private practice. In Austria the veterinary school was founded at Vienna by Maria Theresa, and completed by the Emperor Joseph II, essentially as a military institution—which it is to-day—under the direction of the Department of War. The discipline is strictly military, and the destination of the greater percentage of the students is the army. In Russia and Italy the veterinary schools are classed more closely with the general educational institutions, but both have a majority of military students. In the former country the same system of free education for future military service, that exists in Prussia, is in vogue. It is only within the last half century that the army veterinarians of Europe have been commissioned officers. In Prussia the veterinarians enter the army as non-commissioned officers, and after two years receive the commission of lieutenant, but it must be remembered that the army surgeon, in the same country, only . ecame a commissioned officer in 1845. In all other civilized countries, except the United States, the veterinarian enters as a lieutenant and may be promoted to the rank of colonel. In France the veterinarians became officers with a definitely organized corps in 1843. During the several years previous to this, the loss of horses in the French army was from 80 to 85 per 1000; in 1845, it fell to 77 per 1000. In 1852 the service was improved by increase of pay, pension and retirement, securing better veterinarians, and the annual loss fell to 67 per 1000; in 1860, considerable increased pay again attracted better men and the loss fell to 28 per 1000. In England while custom has kept the hands of the government off, of the veterinary schools at home, they have found it to their advantage to establish and support them as a part of the army in India, where the cavalry is the principal arm. In continental Europe the veterinary student receives a certain amount of instruction in equitation and other practical matters, but on leaving the school for the army he is first sent, for a year or more, to the large cavalry schools and remount stations to familiarize himself with the practical methods of purchase, breaking, training, feeding, shoeing, etc., and for a course of general instruction given to the other officers, before he is assigned to his regiment and assumes great responsibility himself. This "post-graduate" course given at the Cavalry School of Application, (at Saumur) in France, prohibits repetition of the theoretical courses which were taught at the Veterinary Schools. It consists of

1. *Legislation and administration of Military Veterinarians.*—*a.* History of Military Veterinary Medicine. *b.* Study of Regulations. *c.* Reports, etc.

2. *Exterior of Horse.*—*a.* Types, aptitudes, identification. *b.* Domestic and foreign races, adaptibility to cavalry, artillery, wagons, pack, etc. *c.* Practical handling in above.

3. *Military Veterinary Hygiene.*—*a.* Stables, food, harness, work in garrison, field, and transportation by railroad or boat. *b.* Inspection of forage.

4. *Military Veterinary Pathology and Epizootics.*—(Restricted Course) *a.* Diseases peculiar to new horses. *b.* Causes of epizootics in service. *c.* Emergency, treatment in field.

5. *Military Farriery*—*a.* Special shoeing required by different arms of service and seasons of year. *b.* Shoeing for defects of gait.

6. *Clinics.*

7. *Instruction relative to the inspection of Butcher's Meat.*—*a.* Examination of animals on hoof, from sanitary point of view. *b.* Recognition of species of animals, part it comes from in dressed meat. *c.* Quality of meat.

The veterinarian is then assigned to his regiment where, according to his grade, he is placed in charge of the veterinary *personnel*, farriery, veterinary hospitals, forage, examination of horses furnished for remounts, examination of civil veterinarians where needed, etc. The system in Austria, Italy, and Russia is practically the same. In all these countries, the army veterinarian is called on to furnish his services, not only directly to the selection, feeding, care and shoeing of well horses and treatment of ill-ones, and the examination and inspection of the animals and meat which are used as food by the soldiers, but they become, also in a way, officials in the interest of agriculture. If outbreaks of plagues or contagious diseases occur in a community where they are stationed, they are detailed to investigate it, in the interest of protecting the neighboring country from its spread. They form a body of experts to whom the local civil veterinarians can refer.

In the United States we have been slow to recognize the value of the veterinarian to the owners of the millions of dollars invested in live-stock, whether in the hands of the individual, the great industries, or the government. We were a century behind the rest of the civilized world in establishing schools for the education of the veterinarian, and, when these were started, it was left to the honesty of purpose of private individuals to formulate the course of instruction, and it may be fairly claimed for the veterinary schools of this country, that, at the expense of their own pockets, they give a better education than the public ask for, and, a far more thorough one, than the great majority of people have any idea of. We are a century behind all other countries in establishing an Army Veterinary Service. During the war of the rebellion, when hundreds upon hundreds of thousands of horses and mules were needed, and used for government work, a fair attempt was made to employ competent veterinarians, but they existed then in too small numbers to be of value except in restricted localities. A number of graduates from England and other countries, and a few self-educated men found employment, and gave valuable assistance as civil employes of the Quartermaster's Department, but few found their way to the field. After the horses and mules which had cost millions of dollars had left the supply depots, they were left to the mercies of the most skillful, and compassionate horseman in the troop to which they belonged, or to the ignorant brutality of that class of pushing stablemen who find employment as healers of *horses*, from otherwise intelligent men, who would not employ equally ignorant and assuming laborers to treat their dogs or to sweep out their offices. Thousands of horses died of simple troubles which even the hardship of active service did not demand; thousands were abandoned or destroyed for foot troubles, which might have been remedied on a march by proper treatment; glanders, a curse of war in all countries, was allowed to spread to an extent unheard of since the long wars in Germany, from want of system and government authority, for, while many commanders recognized the disease and destroyed the infecting animals, the neighboring commander did not, and the sacrifice of the first was unavailing. Again, many cases of distemper were destroyed from error in diagnosis. Toward the end of the war, as contract employes, the veterinarians had in many places become respected advisers in the army, and a golden opportunity was lost of establishing a definite corps.

The following taken from a paper by Dr. Olof Schwartzkopff, veterinary surgeon Eighth U. S. Cavalry, gives actually the present condition of the veterinary surgeons now employed in the army:—

"The source from which army veterinary surgeons have been appointed "for many years was, for the most part, from army farriers without other "qualfications than an ever-readiness in dosing our animals with strong smell- "ing horse medicines. The first step in the right direction, to establish an "army veterinary service on a more professional basis, was taken in October, "1878, when a board of officers were convened to prepare and recommend "a standard supply table of veterinary medicines and instruments for the "use of the army. Among other resolutions it is said in Paragraph III, as "follows:—

"Hereafter appointments as veterinary surgeons will be confined to the "graduates of established and reputable veterinary schools and colleges. "They will be appointed by the Secretary of War in number, etc., etc.

"This showed, indeed, a tendency to reform, but at the same time no "professional organization of the army veterinary surgeons took place, their "duties and privileges remained uncertainly defined and they were left to "themselves without communication or professional direction. The medi- "cines provided by the standard supply table were well selected and abund- "ant, but in the hands of the ignorant troop farrier they became dangerous, "misleading them to quackery and to the torture of the unfortunate horse "with the furnished drenching horn, an instrument which really belongs "in a veterinary museum.

"This situation soon became known in cavalry circles and as a result "a new board on cavalry equipments on veterinary supply table for the "army was convened in April, 1884, consisting of four cavalry officers—but "no veterinary surgeon—to reduce this supply of veterinary medicines. 'This board among its other restrictions says:—It is a necessity of giving

"the uneducated farrier as few medicines as possible. That is very true, "but as medicines should be properly for the use of the veterinary surgeon "and dispensed only under his directions, nevertheless attention was paid to "him in a very brief manner, by recommending that for the practice of the "veterinary surgeon, additional articles can be obtained by approval of post "and department commanders, with such other restrictions as may be re-"quired. This decision is to the veterinary surgeon of no value, as it is "known from previous experience, that it will require two or three months "after the requisitions have been forwarded to obtain the required medicines. "This places the educated veterinary surgeon on the same footing as the "ignorant farrier, as it prescribes him only thirty-one medicines for use in "his professional treatment.

"Considering that after a reduction was made by the board in the "veterinary supply table, the veterinary medicines purchased from the "medical department amounted to $13,332.02, (report of the Q. M. General, "1885).[1] The sum is extremely large and could be reduced to one-third, if "not less by judicious selection and requisition, made by the veterinary "surgeon.

"The same board of cavalry officers decreased the supply of instruments. The pocket-cases, post-mortem cases, etc., etc., furnished according "to the supply table of 1879, were cancelled in 1884, and a very limited unpro-"fessional selection of single surgical knives and scissors substituted. As "the veterinary instruments are kept by the post quartermaster, the vete-"rinary surgeon is debarred from their care and use ; he has to borrow them, "when required, which causes an unnecessary delay and sometimes they "cannot be had through the absence of some employé on Sundays or holi-"days.[2] These instruments, having no professional care, become unservice-"able in a short time; and if an operation of any importance has to be per-"formed the veterinary surgeon is compelled from these causes, to use his "private instruments, which is certainly not the intention of the United "States Government.

"Finally, the board, in 1884, furnished only two veterinary books : Flem-"ing's Horse Shoeing, a small but competent book, and Farmer's Veteri-"rinary Adviser, by Law, which may be very useful in the sphere it implies, "but for professional veterinary surgeons is about as serviceable as a family "medical vade-mecum would be to our army surgeons.

"This veterinary supply table recommended by the board in 1884, is "legal up to this date.

"If we now review the professional position of the veterinary surgeon, "we find that he has but little support in regard to his duties and privileges. "Very little is said about it in the army regulations and cavalry tactics, and "this with open cautiousness. This puts the veterinary surgeon in a doubtful "place and his position is dependent by favor or disfavor of his commanding "officer and his special orders in regard to veterinary matters. No office or "veterinary hospital is given him ; he is compelled to go to the different

[1] In 1888 the cost was reduced to $4,506.64.—Ed.

[2] The cause for this statement must have had some unfortunate origin as most commanders allow full control of instruments to the veterinary surgeon.—Ed.

"stables for the treatment of the sick animals. The gathering together and "carrying of implements from one stable to another in special cases, causes "trouble and delay, and much treatment must be omitted by want of proper "medicine, instruments and any arrangement for certain diseases. The vet-"erinary surgeon gets but little willing assistance from the farrier and shoe-"ing-smith, who logically should be his subordinates. But he has no "authority over them, his instructions are carelessly executed and his orders "in the treatment and shoeing of horses often changed by others in authority, "who measure their competence to indulge in the practice of scientific medi-"cine among horses by virtue of their rank, which the veterinary surgeon "does not possess.

"Now the responsibility of the troop commander over his horses under "treatment of a veterinary surgeon is a great drawback, as it prevents the "latter from using his judgment and energies in critical cases, for it is "almost certain, that a difference of opinion may exist, and all exact knowl-"edge and practice gained by years of study in colleges and universities "goes to naught by reason of prejudice and superstition. But cavalry tactics "require officers that they shall be able to treat all ordinary cases of injury "and disease in horses. This is exactly the point in view of one hundred "years ago, but to-day is an absurdity. The only competent authority to "treat disease in horses is the veterinary surgeon, and he alone should be "responsible for the proper medical treatment But the general rules of the "hygienic care of horses in their natural condition, the knowledge of hip-"pology, should be well known to every cavalry officer. To be a good horse-"man is a necessary qualification of a cavalry officer, and gives him a wide "field of studies, as hippology contains : The history ot the horse, the his-"tory of the art of breeding, training, saddling, riding , also the sanitary "care and economy of the horses in regard to the principles and practice of "stabling, feeding, watering, grooming, and finally the knowledge of the "exterior conformation of the horse on a general anatomical basis with the "view of proper judgment for the purchase of horses to the different branches "of the service. But quite separate from modern hippology is veterinary "medicine and surgery ; that is absolutely a medical science of vast extent."

But, however good the horseman, and however well grounded he may be in the above subjects, however expert he may be in judging the general conformation and character of a horse, his adaptability to cavalry, light battery or quartermaster's train, he cannot have the subtle acuteness to detect slight blemishes, which will develop into grave diseases, signs of anatomical weakness and physiological abberations which will render an animal unsound,and pathological lesions which depreciate his value, unless he has the knowledge of anatomy, physiology and pathology which is possessed by the veterinarian. The latter has gained this knowledge by years of hard study, and is the proper expert to be always employed as one of a board of purchase.

You learn daily the evil effect which faulty shoeing has on the entire leg, to the shoulder in front and to the croup behind. No one but the veterinarian with your education is competent to supervise farriery, and to protect both in health and disease the most important part of the horse, the foot, for no one else can detect the threatened diseases, which are being caused, until the evil has been done.

From the Quartermaster General's report for 1888, we find that there were in the United States Army for the preceding year the following animals :—

*Number of Animals U. S. Army.*

| | Cavalry and Artillery Horses. | Team Horses. | Mules. | Oxen. |
|---|---|---|---|---|
| On hand . . . . . . . . . . | 6.241 | 355 | 5,326 | 6 |
| Purchased and taken up . . | 928 | 30 | 14 | 0 |
| Total . . . . . . . . . . | 7,169 | 385 | 5,340 | 6 |

Total animals, 12,900.

*Value of Animals Purchased.*

| | No. | Average cost. | Total cost. |
|---|---|---|---|
| For Cavalry and Artillery . . . | 924 | $137.59 | $127,138.35 |
| Team horses . . . . . . . . . | 30 | 201.73 | 6,052.00 |
| Mules . . . . . . . . . . . . | 5 | 180.00 | 700.00 |
| Total . . . . . . . . . . . . | 959 | . . . | $134,090.35 |

If we will take the above prices as value required to supply the entire army, we find that the amount invested by the Govment, is :

| | No. | Value. |
|---|---|---|
| Cavalry and artillery horses . . . . . | 7,169 | $985,737.50 |
| Team horses . . . . . . . . . . . . | 355 | 77,666.00 |
| Mules . . . . . . . . . . . . . . . | 5,340 | 961,200.00 |
| Oxen . . . . . . . . . . . . . . . | 6 | 300.00 |
| Total . . . . . . . . . . . . . . | 12,900 | $2,024,903.50 |

There were, died and sold, the latter presumably for loss of usefulness :—

| | No. | Cost at above values. |
|---|---|---|
| Cavalry and artillery horses . . . . . | 1,128 | $155,100.00 |
| Team horses . . . . . . . . . . . . | 68 | 13,717.64 |
| Mules . . . . . . . . . . . . . . . | 700 | 126,000.00 |
| T tal . . . . . . . . . . . . . . | 1,896 | 294,817.64 |
| Amount received from sale of horses and mules . . . . . . . . . . | . . . | 72,108.09 |
| Loss, death and sale . . . . . . . . | | $222,709.55 |

In round numbers we have a cavalry of over 12,000 horses and mules, valued at over $2,000,000, and sustained an annual loss of 15 per cent. which must be replaced. Look at it from a business basis, and certainly such an investment of capital demands a proper protection.

For some years the veterinarians of the army and the United States Veterinary Medical Association, and liberal officers of the United States Army, have endeavored to obtain a change in the present system and an improvement in the status of its representatives. A number of bills have been formulated and some even have been presented in Congress. All have striven toward the same objective point, but from faults of misunderstanding, over zeal, or want of method, have died at an early period of their career. All have, however, acted as a leaven in promoting the object for which they were originated.

The late distinguished Commander of the United States Army, Lieutenant-General Sheridan, took an active interest in all that pertained to the effective condition of the troops whom he commanded, and for the last two years of his life lent a willing ear to the requests for improvement of the army veterinary service. He had given a generous and liberal attention to the subject when brought before him, and had, late in the winter of 1877–78, reviewed the various proposed acts for the establishment of a veterinary corps and outlined a foundation which he proposed to recommend in his next annual report, and which he would have seen succeed had we been spared his loss. His successor, General Schofield, was equally progressive in the consideration of the subject when time permitted him to consider it, too late, however, for any action to be taken by the Congress of 1888–89.

I believe, that the following proposition will satisfy the veterinarians throughout the country, in their advocacy and desire to improve the status of their profession, by having an official military recognition, and that it will meet with the endorsement and liberal support of the General of the Army and of the prominent officers of cavalry, artillery, and staff departments of the army. With the support of the latter and an honest exposition of the subject to the stock-raisers and agriculturalists of the country, I believe that we may consign ourselves with safety to our representatives in Congress for a generous vote which will establish an army veterinary service, consisting of a Chief with the rank of a Major of Cavalry, four veterinarians with the rank of Captains of Cavalry, ten assistant veterinarians with the rank of 1st Lieutenants of Cavalry, and ten assistant veterinarians with the rank of 2d Lieutenants of Cavalry. With this nucleus, appointed by a competitive examination, before a board, consisting of the Chief, two officers of the medical department, and two officers of cavalry, which would insure their capability and qualifications, both from a professional and practical standpoint, we would have a corps of officers who would save the government many times the cost of their salaries annually, and would soon make themselves so esteemed that it would be wondered why they were not organized before.

